# Association between the weight-adjusted waist index and stroke: a cross-sectional study

**DOI:** 10.1186/s12889-023-16621-8

**Published:** 2023-09-01

**Authors:** Jiayi Ye, Yanjie Hu, Xinrong Chen, Zhe Yin, Xingzhu Yuan, Liping Huang, Ka Li

**Affiliations:** West China Hospital, Sichuan University/West China School of Nursing, Sichuan University, Chengdu, 610041 China

**Keywords:** Weight-adjusted waist index, Stroke, Obesity, NHANES, Cross-sectional study

## Abstract

**Background:**

The weight-adjusted waist index (WWI) is a new measure of obesity, and this study aimed to determine the association between the WWI and stroke.

**Methods:**

Using the National Health and Nutrition Examination Survey (NHANES) 2011–2020 dataset, cross-sectional data from 23,389 participants were analysed. The correlation between the WWI and stroke was investigated through multivariate logistic regression and smoothing curve fitting. Subgroup analysis and interaction tests were also carried out.

**Results:**

The research involved 23,389 participants, of whom 893 (3.82%) had a stroke. The fully adjusted model revealed a positive correlation between the WWI and stroke [1.25 (1.05, 1.48)]. Individuals who were in the highest quartile of WWI exhibited a 62% higher likelihood of experiencing a stroke than those in the lowest quartile [1.62 (1.06, 2.48)]. Subgroup analysis and interaction tests revealed that this positive correlation was similar in different population settings (all P for interaction  > 0.05).

**Conclusion:**

A higher WWI was associated with a higher prevalence of stroke. The results of this study underscore the value of the WWI in stroke prevention and management.

## Introduction

Stroke is a cerebrovascular illness characterized by blood vessel obstruction [1]. Stroke was reported to be ranked second globally among the most common causes of mortality and third among the leading causes of death and disability combined [2, 3]. With the ageing population, the frequency of stroke incidence continues to escalate [4]. Specifically, the occurrence of stroke surged by 70%, and its global prevalence rose by 85% between 1990 and 2019 [2]. The high rates of morbidity, mortality, and disability linked to stroke implicate it as a serious threat to public health [5]. Furthermore, the estimated annual global expenditure related to stroke exceeds $89.1 billion, exerting an enormous socioeconomic impact and placing a significant strain on health care systems [6, 7]. As a result, it is necessary to identify preventable and controllable factors to lower the prevalence of stroke.

The prevalence of obesity has reached epidemic levels globally, affecting an increasingly large number of individuals [8–10]. Obesity has been associated with the onset of various diseases, including diabetes, nonalcoholic fatty liver disease, cardiovascular disease, and several malignancies [11–14]. Body mass index (BMI) and waist circumference (WC) serve as common obesity evaluation metrics. However, those indicators cannot be used to differentiate between fat and muscle mass [15–17]. Recent studies have proposed that body composition and fat distribution can be used to more accurately assess poor metabolic characteristics [18, 19]. The weight-adjusted waist index (WWI), a new type of obesity index, standardizes WC with weight, incorporating the strengths of WC while dampening the connection with BMI [20]. The WWI not only differentiates between fat and muscle mass but also accounts for central obesity issues unrelated to weight [21, 22]. Previous research has demonstrated that an elevated WWI is closely linked to several diseases, such as abdominal aortic calcification, osteoporosis, and heart failure [23–25].

The etiopathogenesis of stroke is complex, and obesity is considered to be one of the major risk factors [26]. While the WWI serves as an effective predictor of risk for numerous cardiovascular diseases, no studies to date have explored a potential relationship between the WWI and stroke. Therefore, we aimed to examine this relationship through a cross-sectional analysis using data from the 2011–2020 National Health and Nutrition Examination Survey (NHANES).

## Methods

### Survey description

NHANES is a continuous US health investigation run by the Centers for Disease Control and Prevention (CDC) [27]. To accurately evaluate the health and nutritional status of the US population, the survey employed stratified multistage probability sampling to generate a representative sample [28]. An in-home interview, physical examination, and a battery of laboratory tests were performed on all participants.

The study procedure was approved by the National Center for Health Statistics (NCHS) Research Ethics Review Board. Before the commencement of research, every participant furnished written permission [29]. The comprehensive depiction of the NHANES research and its corresponding statistics can be found at https://www.cdc.gov/nchs/nhanes/.

### Study population

**Fig. 1:**
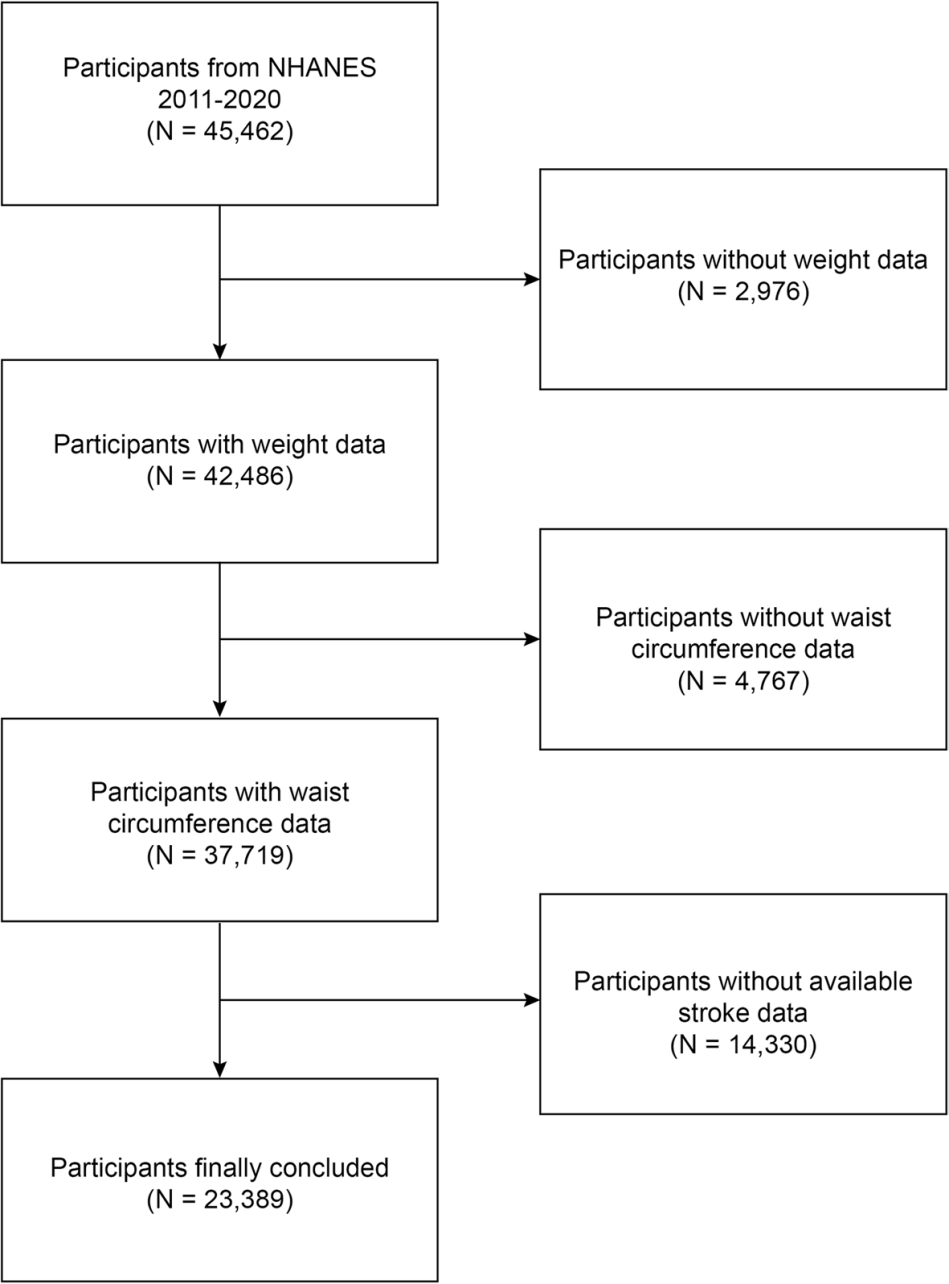
Flow chart of participant selection. NHANES, National Health and Nutrition Examination Survey

This investigation employed the NHANES dataset from 2011–2020. Participants with complete stroke and WWI data were included in our study. Initially, 45,462 participants were recruited. After eliminating participants with absent or incomplete data concerning weight (*n* = 2,976), WC (*n* = 4,767), and stroke (*n* = 14,330), our final analysis included 23,389 participants (Fig. 1).

### Weight-adjusted waist index

The WWI is a new obesity assessment indicator. A greater WWI score indicates a higher level of obesity. The WWI (cm/√kg) is calculated by dividing WC (cm) by the square root of weight (kg) [20]. Anthropometric measurements were taken at a mobile examination centre by skilled medical personnel and recorded by professional recorders to ensure the accuracy of the data. Digital scales were used for weight measurement. Participants wore examination clothing prior to weighing and subsequently stood barefoot in the middle of a digital scale with their arms next to their bodies and their eyes gazing forwards [23]. Tape measures were used for WC measurement. A tape measure was positioned at the junction of the mid-axillary line and a horizontal line above the uppermost lateral edge of the right patella [24]. Our research considered the WWI to be an exposure variable.

### Stroke

A medical conditions questionnaire was used to determine the occurrence of stroke. Participants were judged to have experienced a stroke if they replied "yes" to this question: "Has a doctor or other health professional ever told you that you had a stroke?" Previous research has validated the use of self-reported stroke [30, 31]. In our study, stroke was regarded as an outcome variable.

### Covariates

In the present study, we included covariates that could obfuscate the relationship between the WWI and stroke, including age, sex, race, education level, smoking, diabetes, high blood pressure, coronary heart disease, cancer, ratio of family income to poverty (PIR), average alcohol consumption in the past 12 months, triglycerides, high-density lipoprotein cholesterol (HDL-C), low-density lipoprotein cholesterol (LDL-C), and total cholesterol (TC).

### Statistical analysis

Statistical analyses conducted for this study were executed in compliance with guidelines provided by the CDC, with proper NHANES sample weights, and taking into account complex multistage clustering surveys. The research assessed the characteristics of participants based on quartiles of the WWI utilizing chi-square tests or t-tests. Multivariate logistic regression was used to examine the linear association between the WWI and stroke in three different models. Model 1 did not involve any covariate adjustments. Model 2 involved the adjustment of age, sex, and race. Model 3 adjusted for age, sex, race, education level, alcohol consumption, smoking, diabetes, high blood pressure, coronary heart disease, cancer, PIR, triglycerides, TC, HDL-C, and LDL-C. After converting the WWI score from continuous to classified variables (quartiles), trend tests were applied to explore trends in the linear correlation between the WWI and stroke. Subgroup analyses of the relationship between the WWI and stroke were performed using stratifying factors, comprising age, sex, race, education level, diabetes, high blood pressure, coronary heart disease, and cancer, and interaction tests were used to test the consistency of this association within distinct groupings. Moreover, smoothing curve fitting was conducted to investigate a nonlinear correlation between the WWI and stroke [32]. We carried out all statistical analyses using R (version 4.2) and Empower software (version 5.0). A two-sided *p* < 0.05 was considered to indicate a statistically significant difference.

## Results

### Baseline characteristics

The study involved 23,389 participants with an average age of 49.32 ± 17.42 years, of whom 11,409 (48.78%) were male and 11,980 (51.22%) were female. Among all participants, the prevalence of stroke was 3.82%, and it increased with the higher WWI quartiles. The mean WWI for all participants was 11.09 ± 0.86 cm/√kg, with the values for the different quartiles as follows: quartile 1: < 10.51; quartile 2: 10.51–11.09; quartile 3: 11.10–11.67; and quartile 4: > 11.67 cm/√kg. Compared to those with the lowest quartile of WWI, individuals with the highest quartile of WWI were more likely to be older, female, non-Hispanic white, less educated, and smokers, and they were more likely to have diabetes, high blood pressure, coronary heart disease, cancer, and stroke. Meanwhile, a higher WWI tended to be accompanied by a higher BMI, WC, body weight, triglyceride, LDL-C, and TC levels, while PIR and HDL-C levels were lower (Table 1).

**Table 1:** Basic characteristics of participants by weight-adjusted waist index quartile

Characteristics	Weight-adjusted waist index				*P* value
	**Q1 (< 10.51)** ***N* = 5,847**	**Q2 (10.51–11.09)** ***N* = 5,847**	**Q3 (11.10–11.67)** ***N* = 5,847**	**Q4 (> 11.67)** ***N* = 5,848**	
Age (years)	37.06 ± 13.45	46.33 ± 15.05	51.74 ± 15.90	57.47 ± 16.14	< 0.001
Sex, (%)					< 0.001
Male	59.02	52.33	46.07	32.29	
Female	40.98	47.67	53.93	67.71	
Race/ethnicity, (%)					< 0.001
Non-Hispanic White	64.39	64.73	64.07	67.08	
Non-Hispanic Black	14.97	10.25	10.01	9.27	
Mexican American	5.58	8.57	10.60	9.91	
Other race/multiracial	15.06	16.45	15.32	13.74	
Education level, (%)					< 0.001
Less than high school	9.01	12.32	16.07	19.36	
High school	18.20	21.31	24.15	26.44	
More than high school	72.79	66.33	59.76	54.17	
Smoking, (%)					< 0.001
Ever	36.91	43.59	46.87	47.93	
Never	63.09	56.41	53.13	52.07	
Diabetes, (%)					< 0.001
Yes	3.01	8.60	14.85	26.76	
No	96.99	91.40	85.15	73.24	
High blood pressure, (%)					< 0.001
Yes	13.89	29.05	38.80	52.73	
No	86.11	70.95	61.20	47.27	
Coronary heart disease, (%)					< 0.001
Yes	0.83	2.23	4.28	7.20	
No	99.17	97.77	95.72	92.80	
Cancer, (%)					< 0.001
Yes	5.09	9.75	11.87	16.89	
No	94.91	90.25	88.13	83.11	
Stroke, (%)					< 0.001
Yes	0.90	2.00	3.08	5.49	
No	99.10	98.00	96.92	94.51	
BMI (kg/m^2^)	24.96 ± 4.70	28.32 ± 5.32	30.67 ± 6.37	34.22 ± 7.82	< 0.001
Waist circumference (cm)	86.09 ± 10.56	97.24 ± 11.61	104.79 ± 13.19	115.34 ± 16.13	< 0.001
PIR	3.08 ± 1.62	3.14 ± 1.59	2.96 ± 1.60	2.61 ± 1.52	< 0.001
Weight (kg)	74.70 ± 16.85	82.06 ± 19.41	86.29 ± 21.81	91.65 ± 25.21	< 0.001
Average alcohol consumption past 12 months	3.24 ± 18.67	4.29 ± 39.96	2.99 ± 20.49	3.33 ± 29.64	0.187
Triglycerides (mg/dL)	107.49 ± 63.03	117.45 ± 67.70	124.53 ± 80.28	126.97 ± 61.62	< 0.001
HDL-C (mg/dL)	57.27 ± 16.23	54.00 ± 16.65	52.22 ± 16.84	51.20 ± 14.41	< 0.001
LDL-C (mg/dL)	109.30 ± 22.37	113.27 ± 23.74	113.47 ± 24.32	111.61 ± 24.87	< 0.001
Total cholesterol (mg/dL)	183.55 ± 37.46	194.32 ± 39.38	196.08 ± 42.12	192.35 ± 42.86	< 0.001

Mean ± SD for continuous variables: the P value was calculated by the weighted linear regression model

(%) for categorical variables: the P value was calculated by the weighted chi-square test

*Abbreviations*: *Q* Quartile, *BMI* Body mass index, *PIR* Ratio of family income to poverty, *HDL-C* High-density lipoprotein cholesterol, *LDL-C* Low-density lipoprotein cholesterol

### Association between the WWI and stroke

**Fig. 2:**
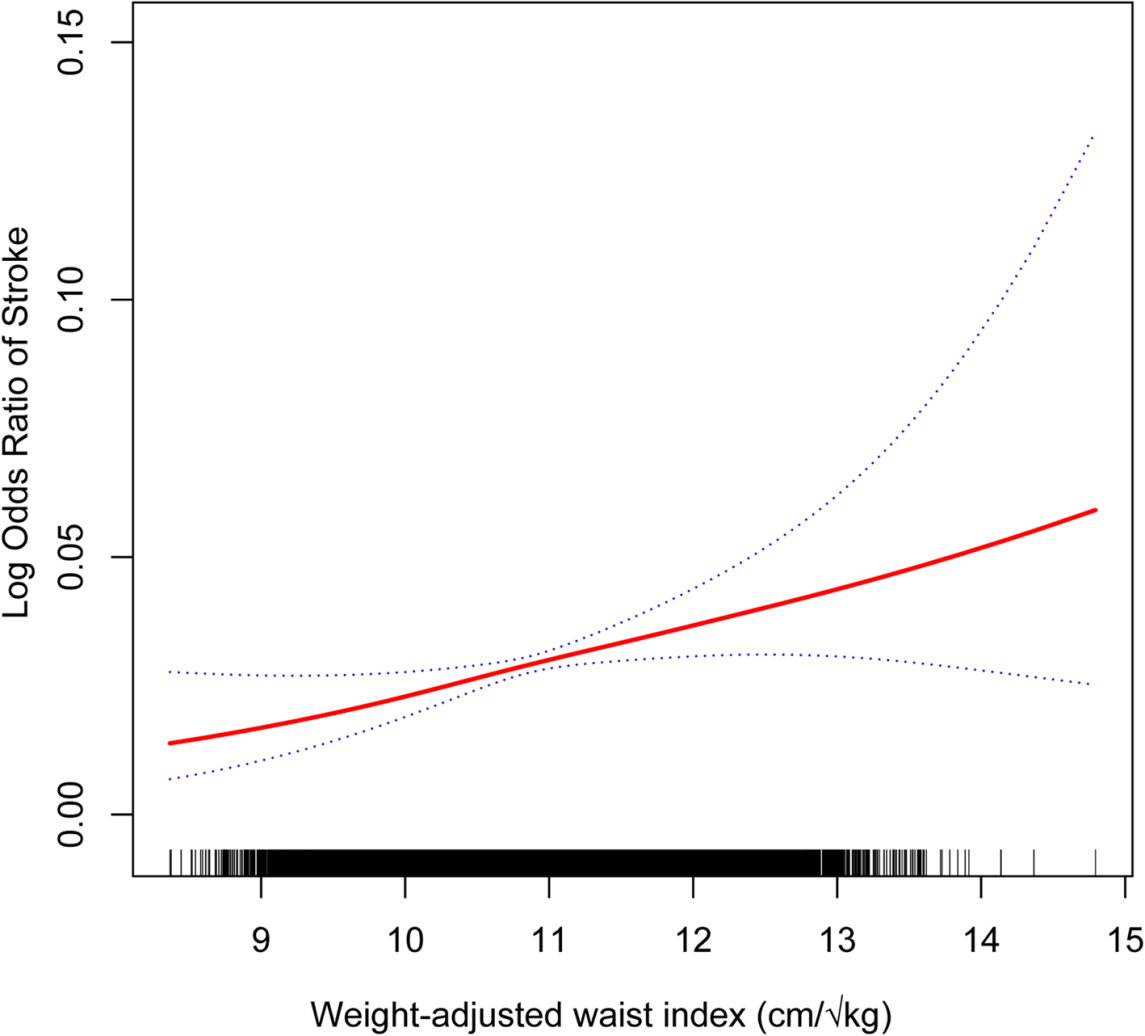
The nonlinear association between the WWI and stroke. The solid red line represents the smooth curve fit between variables. Blue bands represent the 95% confidence interval from the fit. WWI, weight-adjusted waist index

Table 2 shows the correlation between the WWI and stroke. In the crude [1.94 (1.79, 2.10)] and partially adjusted [1.36 (1.23, 1.50)] models, the WWI and stroke showed a significant positive association. Upon complete adjustment, the aforementioned positive association remained statistically significant [1.25 (1.05, 1.48)], with a 25% increase in stroke prevalence for every unit increase in the WWI. This positive association remained stable after transforming the WWI into quartiles (all P for trend < 0.05). Participants in the highest WWI quartile had a 62% increased prevalence of stroke compared to those in the lowest quartile [1.62 (1.06, 2.48)]. Furthermore, the findings of smoothed curve fitting analysis corroborated the nonlinear positive correlation between the WWI and stroke (Fig. 2).

**Table 2:** The association between weight-adjusted waist index and stroke

Exposure	Model 1 [OR (95% CI)]	Model 2 [OR (95% CI)]	Model 3 [OR (95% CI)]
Weight-adjusted waist index (continuous)	1.94 (1.79, 2.10)	1.36 (1.23, 1.50)	1.25 (1.05, 1.48)
Weight-adjusted waist index (quartile)			
Quartile 1	reference	reference	reference
Quartile 2	2.12 (1.61, 2.79)	1.34 (1.01, 1.78)	1.36 (0.93, 1.99)
Quartile 3	3.56 (2.75, 4.60)	1.70 (1.30, 2.23)	1.35 (0.91, 1.99)
Quartile 4	5.35 (4.18, 6.86)	2.05 (1.56, 2.69)	1.62 (1.06, 2.48)
P for trend	< 0.001	< 0.001	0.038

Model 1: no covariates were adjusted. Model 2: adjusted for age, sex, and race. Model 3: adjusted for age, sex, race, education level, alcohol consumption, smoking, diabetes, high blood pressure, coronary heart disease, cancer, PIR, triglycerides, total cholesterol, HDL-C, and LDL-C

*Abbreviations*: *PIR* Ratio of family income to poverty, *HDL-C* High-density lipoprotein cholesterol, *LDL-C* Low-density lipoprotein cholesterol

### Subgroup analyses

We performed subgroup analyses and interaction tests stratified by sex, age, race, education level, diabetes, high blood pressure, coronary heart disease, and cancer to evaluate the robustness of the relationship between the WWI and stroke and to discover possible population differences (Table 3). Our outcomes showed that the relationship between the WWI and stroke was not dependent on the above factors (all P for interaction  > 0.05). Moreover, the positive correlation of the WWI with stroke was similar across populations with different sex, age, race, education level, diabetes, high blood pressure, coronary heart disease, and cancer status, and it may be appropriate for different populations.

**Table 3:** Subgroup analysis of the association between weight-adjusted waist index and stroke

Subgroup	Stroke [OR (95% CI)]	P for interaction
Sex		0.103
Male	1.39 (1.11, 1.74)	
Female	1.11 (0.90, 1.38)	
Age		0.131
< 60 years	1.72 (1.39, 2.13)	
≥ 60 years	1.41 (1.15, 1.73)	
Race/ethnicity		0.698
Non-Hispanic White	1.39 (1.08, 1.80)	
Non-Hispanic Black	1.13 (0.84, 1.52)	
Mexican American	1.09 (0.54, 2.22)	
Other race	1.34 (0.85, 2.12)	
Education level		0.442
Less than high school	1.55 (1.07, 2.23)	
High school	1.07 (0.78, 1.48)	
More than high school	1.23 (0.96, 1.57)	
Diabetes		0.309
Yes	1.42 (1.06, 1.91)	
No	1.19 (0.97, 1.45)	
High blood pressure		0.163
Yes	1.16 (0.96, 1.41)	
No	1.44 (1.10, 1.88)	
Coronary heart disease		0.173
Yes	1.75 (1.06, 2.90)	
No	1.21 (1.01, 1.46)	
Cancer		0.110
Yes	1.66 (1.13, 2.43)	
No	1.18 (0.97, 1.43)	

Age, sex, race, education level, alcohol consumption, smoking, diabetes, high blood pressure, coronary heart disease, cancer, PIR, triglycerides, total cholesterol, HDL-C and LDL-C were adjusted

*Abbreviations*: *PIR* Ratio of family income to poverty, *HDL-C* High-density lipoprotein cholesterol, *LDL-C* Low-density lipoprotein cholesterol

## Discussion

In this cross-sectional study involving 23,389 representative adults, we discovered a positive association between the WWI and stroke prevalence, signifying that individuals with a higher WWI had an elevated probability of experiencing a stroke. The subgroup analyses and interaction tests confirmed the robustness of this positive association across a diverse array of demographic contexts. These observations suggested that an elevated WWI could be an independent risk factor for stroke, thus underscoring the significance of the WWI in the prevention and management of stroke.

To the best of our knowledge, this is the first study to examine the link between the WWI and stroke. Prior research has investigated the correlation between the WWI and cardiovascular diseases. Ding et al. conducted a prospective analysis with 12,447 Chinese individuals and discovered a nonlinear positive correlation between the WWI and all-cause and cardiovascular mortality, with a WWI > 11.2 cm/√kg substantially increasing the risk of death [33]. Zhang et al. revealed that a greater WWI may be an independent predictor of heart failure in cross-sectional research of 25,509 people [25]. Cai et al. also reported that the WWI was related to left ventricular hypertrophy, indicating that the WWI may serve as a significant predictor of cardiometabolic risk [34]. Furthermore, Li et al. discovered that WWI was related to a higher incidence of hypertension in rural China, which could potentially serve as an indicator of the risk of hypertension among a rural population in China [35]. We identified a positive correlation between WWI and stroke, which was in line with the detrimental consequences of WWI on cardiovascular health described in earlier research.

Accumulating evidence confirms that obesity is a significant stroke risk factor. BMI and WC are traditional indicators of obesity. Kurth et al. analysed data from 39,553 female participants and found that BMI was a significant risk indicator for overall stroke and for ischaemic varieties of stroke [26]. Each unit rises in BMI was related to an increase of 6% in the odds of total, ischaemic, and haemorrhaging stroke, as revealed in prospective research including 21,000 males in the US [36]. A cross-sectional study of 21,749,261 Korean participants by Cho et al. showed a linear positive correlation between WC and the prevalence of ischaemic stroke [37]. Despite growing evidence that these traditional indicators of obesity are associated with stroke, the obesity paradox persists [38]. The underlying cause of the controversy may be attributed in part to the limitations of traditional indicators, which do not differentiate between fat mass and muscle mass [15–17]. WWI, a new obesity indicator, combines the benefits of WC while weakening the association with BMI, accurately indicating central obesity regardless of body weight. Studies have shown that central obesity is strongly associated with the risk of cardiovascular disease [39]. WWI as an indicator of central obesity, may more accurately reflect risk factors associated with stroke, such as metabolic syndrome, diabetes, and hypertension [40–42]. WWI can also be used to evaluate fat and muscle mass regardless of BMI category [21]. As a result, WWI might serve as a more comprehensive and accurate measure of obesity, and it may more accurately show the correlation that exists between obesity and stroke. WWI was found to be the strongest predictor of a wide range of diseases in recent studies, outperforming BMI and WC [23, 43]. Similar to our findings, we suggest that the WWI may be an independent risk factor for stroke. Different from BMI, which focuses only on weight in relation to height, WWI also considers WC, which helps to capture the risk of central obesity. Second, WWI provides a more direct, simple, and specific way to assess central obesity than other metrics associated with fat distribution, such as the waist-to-hip ratio (WHR). And WWI is applicable to different races and populations and may be more stable and reliable, especially in cross-racial or multicenter studies [44]. In summary, WWI is simple to calculate, economical, and practical, with superior performance in predicting disease risk, and deserves the attention of health care professionals.

Several potential mechanisms could account for this positive correlation between the WWI and stroke. First, the increased WWI might be a reflection of malfunction in adipose tissue, thereby promoting the production and release of various proinflammatory cytokines [45]. These cytokines are involved in all stages of the formation, progression, erosion, and rupture of atherosclerotic plaques, which leads to thrombo-embolic events [46–48]. Second, central obesity can increase oxidative stress in individuals, which is closely associated with the development of atherosclerosis [49, 50]. Adipose tissue releases more reactive oxygen species (ROS) in individuals with obesity [51]. Excess ROS reduces the bioavailability of nitric oxide (NO), and superoxide readily reacts with NO to produce harmful hydrogen peroxide, which ultimately results in malfunction of endothelial cells [52]. Moreover, the increase in ROS promotes the oxidation of low-density lipoprotein in atherosclerotic lesions, which promotes immune responses in endothelial cells, including elevated levels of adhesion molecule expression, macrophage migration, and the formation of lipid-containing macrophages [53, 54]. These processes exacerbate damage to the vascular endothelium. Third, other disease states that coexist with obesity, such as impaired glucose tolerance, hypertriglyceridaemia, and high blood pressure, also increase the risk of stroke [55].

The strength of this study is that it was based on NHANES data, which were collected using a stratified multistage probability sampling strategy, thus making the study more reliable and representative. Second, we conducted a subgroup analysis to further clarify the association of WWI with stroke in different population settings. Finally, we adjusted for confounding factors including age, sex, race, education level, alcohol consumption, smoking, diabetes, high blood pressure, coronary heart disease, cancer, PIR, triglycerides, TC, HDL-C, and LDL-C to lessen the impact of confounding and obtain more reliable findings. Nevertheless, this research also presents certain constraints. The causal association between WWI and stroke could not be determined due to limitations inherent in the cross-sectional study design. Furthermore, the stroke inclusion criteria relied on self-reported stroke history, and subtypes of stroke were not clear. We were unable to further assess the association between different stroke subtypes and WWI. And the average age of the participants was 49.32 ± 17.42 years, at this age the risk and prevalence of stroke are low. Moreover, even if some potential confounders had been adjusted for, the effect of other potential confounders could not be completely excluded. However, the existing link between WWI and stroke was robust enough that it was unlikely to be greatly altered by confounders that had not been considered.

## Conclusion

The results of this investigation indicated that a greater WWI was associated with an increased risk of stroke. This finding may provide new insights for the future of stroke prevention and treatment. However, higher-quality prospective studies are needed to corroborate our results.

## Data Availability

Publicly available datasets were analyzed in this study. These data can be found here: https://www.cdc.gov/nchs/nhanes/.

## Abbreviations

WWI: Weight-adjusted waist index
BMI: Body mass index
WC: Waist circumference
NHANES: National health and nutrition examination survey
NCHS: National center for health statistics
HDL-C: High-density lipoprotein cholesterol
LDL-C: Low-density lipoprotein cholesterol
TC: Total cholesterol
PIR: Ratio of family income to poverty
CDC: Centers for disease control and prevention
WHR: Waist-to-hip ratio
ROS: Reactive oxygen species
NO: Nitric oxide
